# The Hidden Risks of Hip Replacement: Unveiling Mortality and Costs in 1.6 Million Patients

**DOI:** 10.3390/healthcare13192531

**Published:** 2025-10-07

**Authors:** Yaron Berkovich, Binyamin Finkel, Assil Mahamid, Hadar Gan-Or, Loai Ahmad Takrori, Yaniv Yonai, David Maman

**Affiliations:** 1 Carmel Medical Center, Haifa 3436212, Israel; yaron.berkovich@gmail.com (Y.B.); yanivyonai@gmail.com (Y.Y.); 2 Technion Israel Institute of Technology, Haifa 2611001, Israel; 3 Department of Orthopedics, Hillel Yaffe Medical Center, Hadera 38100, Israeldr.assilm@gmail.com (A.M.); hadarganor@gmail.com (H.G.-O.); loai2030@gmail.com (L.A.T.)

**Keywords:** Total Hip Arthroplasty, post operative complications, clinical outcomes, NIS, National Inpatient Sample, big data

## Abstract

Methods: Using the most recent pre-COVID National Inpatient Sample (2016–2019), we evaluated inpatient mortality and economic impact after elective primary total hip arthroplasty (THA) across 327,123 cases (1,635,615 weighted discharges).Results: Overall inpatient mortality was 0.04%, but was higher in patients ≥ 80 years (0.15%), with weekend admissions (0.10%), and with surgical delay ≥ 1 day (0.17%). Comorbidities with the greatest mortality association included congestive heart failure and chronic kidney disease (both with markedly elevated odds), and acute in-hospital complications (e.g., pulmonary embolism) carried substantial risk. Complications also increased resource use; for example, heart failure, pulmonary edema, and acute coronary artery disease were each associated with significantly higher costs and prolonged length of stay. Conclusion: These findings provide a contemporary, pre-pandemic national baseline that quantifies high-risk subgroups and the economic footprint of adverse events, supporting targeted perioperative strategies and hospital planning for elective THA.

## 1. Introduction

Osteoarthritis (OA) is a prevalent chronic health condition impacting pain, physical function, mental health, sleep, work participation, and even mortality [1]. It stands as the costliest condition for privately insured patients in the United States, contributing to over $6.3 billion in healthcare expenses [2]. With a projected surge in US adults with arthritis expected to reach 78 million by 2040 [3], the economic burden on individuals and the healthcare system is poised to escalate, given that OA is the most prevalent form of arthritis [4].

Primary THAis the gold standard for treating end-stage hip osteoarthritis, providing relief from pain and improved joint function [5]. Its transformative journey began with the first total hip replacement by Wiles in 1938, evolving significantly with Sir John Charnley’s “low-friction arthroplasty” in the 1960s [6,7]. Recognized as “the operation of the century” [7], THA is considered a safe and effective intervention, despite potential side effects such as infection, dislocation, and pulmonary embolism. Inpatient mortality, the most serious complication, has been documented in up to 3.9% of patients after THA [8,9]. While historical literature reports death rates ranging from 0.1 to 0.8% [10], a significant decline can be attributed to technological advancements and improved preoperative treatment [11,12].

This study delves into the national database, exploring variables influencing inpatient mortality rates after hip arthroplasty. The research aims to highlight key findings regarding mortality rates, comorbidities, costs, and other critical parameters, providing valuable insights for the understanding and enhancement of this pivotal surgical intervention. Importantly, our investigation utilizes a more recent dataset (2016–2019) with the ICD-10 coding system, enhancing the relevance and accuracy of our findings compared to an earlier study that employed older data and the ICD-9 coding system [13].

### Research Questions

What are the key predictors of inpatient mortality following primary total hip arthroplasty, and how do comorbidities, surgical delays, and hospital factors influence mortality rates, costs, and length of stay?

## 2. Methods

Our study utilized the National Inpatient Sample (NIS) dataset from 2016 to 2019, the largest all-payer inpatient database in the world. This dataset provides a comprehensive and nationally representative overview of U.S. hospital admissions across various states. Notably, this is the most recent pre-COVID-19 version of the NIS, ensuring that our findings are not confounded by the significant healthcare system disruptions caused by the pandemic.

### 2.1. Data Processing and Cohort Refinement

The raw NIS dataset was systematically filtered to include hospitalizations with ICD-10 procedure codes corresponding to primary total hip arthroplasty. Non-elective, trauma-related, and revision procedures were excluded to ensure a purely elective primary THA cohort. Records with missing or implausible demographic data were also removed. After applying these refinement steps, the final analytic sample comprised 327,123 cases, corresponding to 1,635,615 weighted patient discharges. Age was categorized as <64, 65–79, and ≥80 years to reflect commonly used clinical and health-system thresholds (younger adults, Medicare-age patients, and octogenarians who carry disproportionate perioperative risk).

### 2.2. Definition of Mortality

Inpatient mortality was defined as death occurring during the index hospitalization, based on the NIS variable DIED (coded 1 = died before discharge, 0 = survived). This definition is consistent with prior studies using NIS for perioperative outcomes research [13].

### 2.3. Variables Collected

The dataset included detailed information on demographics, comorbidities, hospital characteristics, and inpatient complications classified by ICD-10 codes. Comorbidities were identified using the Charlson Comorbidity Index and individual ICD-10 diagnostic codes. Hospital characteristics included teaching status, bed size, and geographic region. Socioeconomic status was approximated using income quartile data provided in the NIS. Admission day (weekday vs. weekend) and surgical delay (≥1 calendar day from admission to procedure) were derived from standard NIS fields and treated as categorical exposure variables.

### 2.4. Statistical Analysis

Statistical analyses were performed using SPSS Statistics (version 28) and MATLAB (R2021a). Descriptive statistics summarized patient demographics, comorbidities, and hospital characteristics. Chi-square tests were used for categorical variables, and logistic regression was employed to calculate odds ratios for inpatient mortality. Statistical significance was set at *p* < 0.01. Cost analyses evaluated mean hospitalization costs across comorbidity and complication groups, while length of stay (LOS) was analyzed using ANOVA to assess differences between patient subgroups.

## 3. Results

The study analyzed data from a total of 1,635,615 inpatient THA and found an overall mortality rate of 0.04%. As shown in Table 1, the analysis revealed several statistically significant associations between THA preoperative variables and inpatient mortality. The overall mortality rate was 0.04%. It is noteworthy that the mortality rate increased significantly by 0.15% in patients over 80 years of age. Similarly, the mortality rate for weekend stays was as high as 0.10%. A delay of 1 day or more between admission and surgery was associated with a 0.17% higher mortality rate. Regarding income, the lowest income quartile (0–25%) had a statistically significant mortality rate of 0.06%. In contrast, gender and hospital region did not have a statistically significant effect on inpatient mortality.

Table 2 underscores significant associations between variables and comorbidities with inpatient mortality risk in THApatients. Key findings reveal heightened mortality rates for chronic kidney disease (0.21%) and heart failure (0.30%). Conversely, hypertension (0.02%) is associated with a lower mortality risk compared to the control group (0.06%).

**Figure 1:**
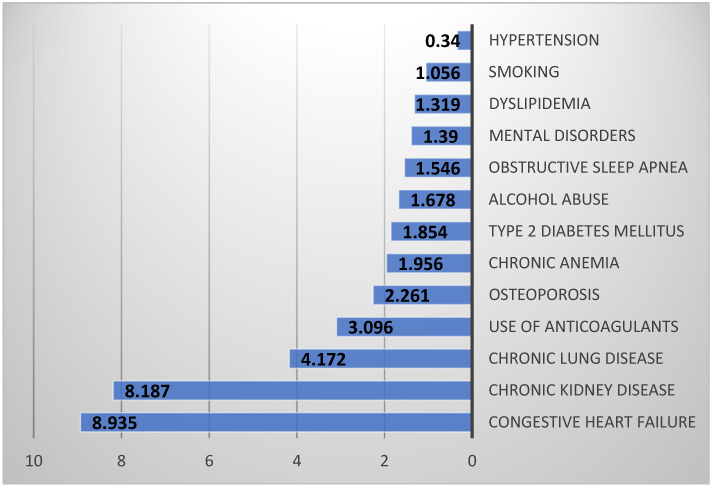
Odds Ratios of Inpatient Mortality After Primary Total Hip Arthroplasty.

Figure 1 displays significant odds ratios for inpatient mortality in primary THA patients, highlighting the relative risks associated with each diagnostic factor. Notably, congestive heart failure and chronic kidney disease present substantially higher odds ratios of 8.935 and 8.187, indicating a significantly increased risk of inpatient mortality.

Hypertension exhibits an odds ratio of 0.34, suggesting a potential association with lower inpatient mortality risk.

Table 3 provides insights into mortality rates segmented by All Patient Refined Diagnosis-Related Group (APR-DRG) Risk Subclasses in elective THA. APR-DRG Risk Subclasses categorize patients based on the predicted risk of mortality, enabling a more nuanced understanding of outcomes. For patients categorized under “Extreme likelihood of dying,” a high mortality rate of 75.40% was recorded, in stark contrast to other risk subclasses. The differences in mortality rates across these APR-DRG Risk Subclasses were statistically significant.

Table 4 outlines hospital complications in elective THA patients, emphasizing diagnostic factors like acute renal failure, exsanguination anemia, and pulmonary embolism, along with their associated mortality rates. For instance, acute renal failure occurred in 1.78% of patients, with a 0.91% mortality rate. Blood loss anemia affected 17.94% of patients, with a 0.11% mortality rate. Although pulmonary embolism is rare (0.08% occurrence), it carries a notably high mortality rate of 3.46%. Statistically significant differences in mortality rates are evident for each inpatient complication type.

**Figure 2:**
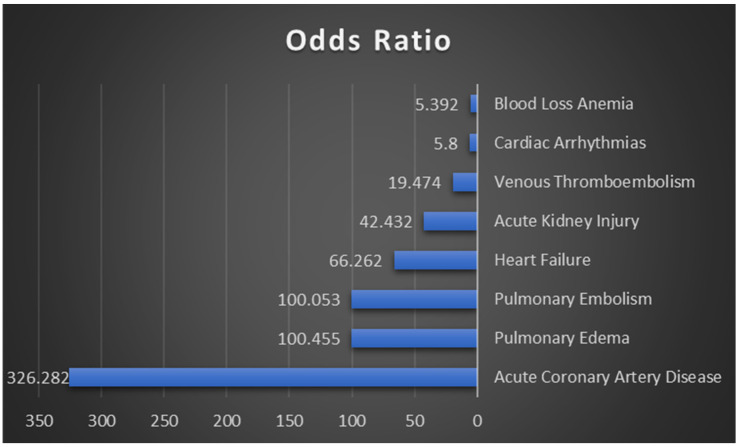
Odds ratios for Inpatient Mortality in Patients Undergoing Primary THA.

Figure 2 depicts odds ratios for inpatient mortality in primary THA patients, emphasizing diagnostic factors. Acute Coronary Artery Disease exhibits the highest odds ratio at 326.282, indicating an exceptionally high likelihood of mortality. Other significant factors include Pulmonary Edema, Pulmonary Embolism, Heart Failure, and Acute Kidney Injury.

Table 5 reveals the influence of comorbidities, complications, and age on average costs for elective THA patients. For instance, acute coronary artery disease substantially raises the average cost from $63,603.94 to $140,793.58. Similarly, patients with heart failure incur an average cost of $132,671.84 compared to $63,605.86 for those without heart failure. The presence of pulmonary embolism and pulmonary edema is also associated with notably higher average costs. Age contributes to cost variation, with patients aged 80 and older incurring an average cost of $66,180.77, surpassing other age groups.

As evidenced by Table 6, the average length of stay in the hospital varied significantly across age categories for patients undergoing elective Total Hip Arthroplasty (THA). Patients aged over 80 had a statistically significant longer average stay of 2.68 days, compared to 2.02 days for those aged 65–79 and 1.9 days for patients under 64.

**Figure 3:**
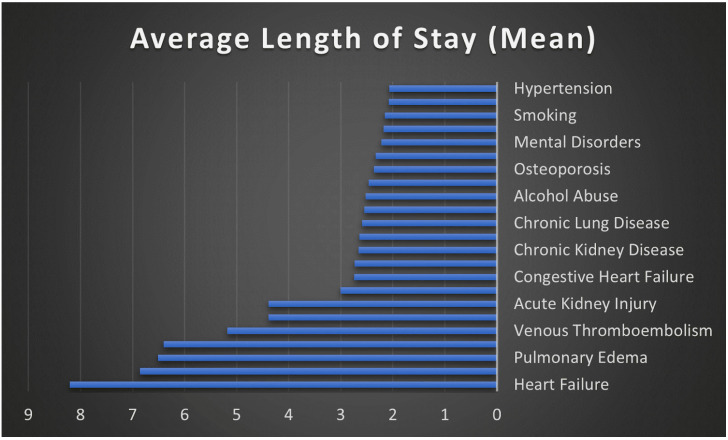
The effect of comorbidities and complications on length of stay in days.

Figure 3 elucidates the statistically significant relationship between various comorbidities and complications and the average length of stay in days for patients undergoing elective THA. Notably, conditions such as acute renal failure and heart failure markedly increased the length of stay to 4.39 and 8.21 days, respectively. Similarly, acute complications like pulmonary embolism resulted in an average stay of 6.86 days.

## 4. Discussion

### 4.1. Contribution of This Study

This national analysis of more than 1.6 million weighted elective THA discharges provides an updated, pre-pandemic benchmark for inpatient outcomes using the NIS (2016–2019) [14]. Our work offers three key contributions: (i) identification of high-risk patient groups (age ≥ 80 years, and those with congestive heart failure [CHF] or chronic kidney disease [CKD]); (ii) demonstration of actionable system-level factors—weekend admission and surgical delay ≥ 1 day—associated with higher inpatient mortality; and (iii) quantification of the economic impact of major complications on costs and length of stay (LOS). Collectively, these results inform perioperative optimization and hospital resource planning in elective THA.

### 4.2. Interpretation of Findings

The overall inpatient mortality rate of 0.04% aligns with contemporary reports of very low perioperative mortality in elective THA and reflects advances in patient selection, perioperative care, and surgical technique [8,9,15]. Nonetheless, the increased mortality among octogenarians (0.15%) and among patients with CHF or CKD underscores the need for tailored risk mitigation and vigilant postoperative monitoring in these populations, consistent with prior literature linking cardiovascular and renal comorbidity to adverse outcomes after arthroplasty [16,17,18]. The large effect sizes observed for acute coronary artery disease, pulmonary embolism, and acute kidney injury reinforce the importance of early recognition and rapid intervention when such complications arise, in line with earlier reports of perioperative cardiac and thromboembolic risk following hip arthroplasty [8,19].

Importantly, our data indicate that system factors remain relevant contributors to adverse outcomes. Weekend admission and surgical delay ≥ 1 day were associated with mortality rates several-fold higher than baseline, suggesting that timely access to surgery and consistent perioperative resources may influence survival. This observation is directionally consistent with prior evidence that organizational and volume-related factors can affect arthroplasty outcomes [8,15].

### 4.3. Economic and Resource Implications

Beyond clinical outcomes, we demonstrate the substantial economic footprint of complications. Heart failure, pulmonary edema, and acute coronary events were associated with markedly higher costs and prolonged LOS. These findings provide quantifiable targets for health-system stakeholders to evaluate the potential value of preoperative optimization, standardized pathways, and early-warning/rapid-response strategies in elective THA, complementing earlier work that connected adverse events with resource utilization after joint replacement [8,9,20].

### 4.4. Clinical Implications

Our findings support practical perioperative strategies for risk mitigation. Patients with CHF and CKD should undergo preoperative optimization including fluid status management, cardiology/nephrology consultation when appropriate, and perioperative hemodynamic monitoring. For patients with high thromboembolic risk (e.g., prior VTE, hypercoagulable states), aggressive thromboprophylaxis and early mobilization protocols are recommended. Hospitals should minimize surgical delay, especially for high-risk patients, by implementing streamlined preoperative clearance pathways and ensuring weekday resource availability.

### 4.5. Comparison with Existing Literature

Our results complement large-scale analyses showing that advanced age and major cardiopulmonary/renal comorbidity are dominant predictors of perioperative risk after hip arthroplasty [13,20,21,22]. The present analysis adds value by focusing on a stable pre-COVID period, thereby avoiding confounding from pandemic-related fluctuations in case mix and hospital operations while providing a national baseline for subsequent comparisons [16]. While procedure volumes varied during and after the pandemic, the patient- and system-level risk patterns identified here remain directly actionable for elective care pathways. Recent large-cohort studies have similarly shown that patient factors such as super-obesity or ethnicity are strongly associated with higher postoperative complication rates, increased cost, and longer hospitalization after THA [23].

### 4.6. Limitations

Several limitations warrant mention. First, our analysis is restricted to inpatient outcomes and does not capture long-term endpoints such as readmissions, functional recovery, or post-discharge survival. The NIS also lacks information on postoperative rehabilitation, patient-reported outcomes, and psychosocial variables (e.g., stress, mental health), which are known to influence recovery trajectories after THA [24]. Although such constraints are inherent to administrative datasets, they limit the ability to fully characterize longitudinal recovery. Future studies that incorporate longitudinal follow-up, rehabilitation utilization, and patient-centered outcomes will be important to provide a comprehensive assessment of long-term clinical and economic impact after elective THA.

## 5. Conclusions

Using the largest pre-COVID nationwide cohort of elective THA cases, we found that inpatient mortality, while rare (0.04%), is concentrated among octogenarians and patients with CHF or CKD. System-level factors such as weekend admission and surgical delay ≥ 1 day were associated with markedly higher risk, identifying actionable targets for process improvement. Complications—including pulmonary embolism, heart failure, and acute coronary events—were not only linked to mortality but also drove substantial increases in cost and length of stay. These findings provide a contemporary baseline for perioperative risk stratification and underscore the economic benefit of preventing complications. Future research should integrate longitudinal follow-up and rehabilitation data to capture the full spectrum of outcomes after THA and guide more comprehensive quality-improvement strategies.

## Figures and Tables

**Table 1:** Mortality Rates in Relation to Pre-Operative Variables for Inpatients Undergoing Total Hip Arthroplasty (THA).

Variable Category	Sub-Category	Mortality (No)	Mortality (Yes)	% Mortality
Age Category	Under 64	666,640	120	0.02%
	65–79	757,640	240	0.03%
	Over 80	137,525	205	0.15%
Gender	Male	729,835	290	0.04%
	Female	904,380	320	0.04%
Region of Hospital	Northeast	328,155	115	0.04%
	Midwest	435,201	155	0.04%
	South	526,625	225	0.04%
	West	344,414	115	0.03%
Income Quartile	0–25%	310,415	185	0.06%
	26–50%	404,360	160	0.04%
	51–75%	440,860	130	0.03%
	76–100%	455,750	120	0.03%
Number of Days from Admission to surgery	0	1,602,055	555	0.04%
	1 or more	32,340	55	0.17%
Admission Day	Weekday	1,629,285	605	0.04%
	Weekend	5110	5	0.10%
TOTAL		1,635,615	610	0.04%

**Table 2:** Impact of Pre-Existing Co-Morbidities on Mortality Rates in Elective Total Hip Arthroplasty Patients.

Co-Morbidity Type	Mortality (No)	Mortality (Yes)	% Mortality
Hypertension			
No	782,195	445	0.06%
Yes	852,200	165	0.02%
Dyslipidemia			
No	942,595	310	0.03%
Yes	691,800	300	0.04%
Sleep Apnea			
No	1,469,875	520	0.04%
Yes	164,520	90	0.05%
Chronic Anemia			
No	1,540,445	545	0.04%
Yes	93,950	65	0.07%
Alcohol Abuse			
No	1,610,200	595	0.04%
Yes	24,195	15	0.06%
Osteoporosis			
No	1,559,155	550	0.04%
Yes	75,240	60	0.08%
Mental Disorders			
No	1,138,690	380	0.03%
Yes	495,705	230	0.05%
Type 2 Diabetes			
No	1,389,950	460	0.03%
Yes	244,445	150	0.06%
Chronic Kidney Disease			
No	1,529,035	390	0.03%
Yes	105,360	220	0.21%
Congestive Heart Failure			
No	1,614,680	550	0.03%
Yes	19,715	60	0.30%
Chronic Lung Disease			
No	1,525,485	470	0.03%
Yes	108,910	140	0.13%
Anticoagulants			
No	1,542,490	515	0.03%
Yes	91,905	95	0.10%

**Table 3:** Mortality Rates by All Patient Refined DRG Risk Subclasses in Elective Total Hip Arthroplasty.

All Patient Refined DRG: Risk of Mortality Subclass	Mortality (No)	Mortality (Yes)	% Mortality
- No class specified	25	0	0.00%
- Minor likelihood of dying	1,357,545	55	9.00%
- Moderate likelihood of dying	236,705	25	4.10%
- Major likelihood of dying	34,890	70	11.50%
- Extreme likelihood of dying	5230	460	75.40%

**Table 4:** Types and Rates of Inpatient Complications in Elective Total Hip Arthroplasty Procedures.

Diagnostic Factor	No (Count)	Yes (Count)	% of All Patients	Died No (%)	Died Yes (%)	% Mortality (No)	% Mortality (Yes)
Acute Renal Failure	1,605,335	29,060	1.78%	345	265	0.02%	0.91%
Blood Loss Anemia	1,341,240	293,155	17.94%	280	330	0.02%	0.11%
Pulmonary Embolism	1,633,095	1,300	0.08%	565	45	0.03%	3.46%
Heart Failure	1,632,895	1,500	0.09%	575	35	0.04%	2.33%
Acute Kidney Injury	1,605,335	29,060	1.78%	345	265	0.02%	0.91%
Acute Coronary Artery Disease	1,633,105	1,290	0.08%	485	125	0.03%	9.69%
Pulmonary Edema	1,633,700	695	0.04%	585	25	0.04%	3.60%
Cardiac Arrhythmias	1,574,670	59,725	3.66%	500	110	0.03%	0.18%
Venous Thromboembolism	1,631,555	2,840	0.17%	590	20	0.04%	0.70%

**Table 5:** Impact of Comorbidities, Complications and Age on Average Costs for Elective Total Hip Arthroplasty Patients.

Condition	Mean Cost in $ (No)	Mean Cost in $ (Yes)
Hypertension	64,032.01	63,339.71
Dyslipidemia	64,054.35	63,147.52
Obstructive Sleep Apnea	63,625.91	64,071.51
Chronic Anemia	63,459.19	67,148.12
Alcohol Abuse	63,601.64	68,310.66
Osteoporosis	63,511.9	66,964.83
Mental Disorders	63,142.14	64,886.35
Type 2 Diabetes	63,280.48	65,889.08
Chronic Kidney Disease	63,286.45	69,265.44
Congestive Heart Failure	63,572.17	71,733.08
Chronic Lung Disease	63,320.64	68,566.06
Use of anticoagulants	63,451.69	67,342.56
Acute Renal Failure	63,158.3	91,788.62
Blood Loss Anemia	61,568.63	73,308.78
Pulmonary Embolism	63,621.45	124,022.33
Heart Failure	63,605.86	132,671.84
Acute Coronary Artery Disease	63,603.94	140,793.58
Stroke	63,670.28	71,158.24
Pulmonary Edema	63,641.96	129,976.01
Cardiac Arrhythmias	63,456.32	69,341.65
Venous Thromboembolism	63,594.89	106,645.06
Age Category (Under 64)		63,717.26
Age Category (65–79)		63,274.75
Age Category (Over 80)		66,180.77

**Table 6:** Influence of Age on Length of Stay.

Age Category in Years	Average Length of Stay in Days
Under 64	1.9
65–79	2.02
Over 80	2.68

## Data Availability

The original contributions presented in the study are included in the article, further inquiries can be directed to the corresponding author.

## Abbreviations

List of Abbreviations (A–Z):

APR-DRG	All Patient Refined Diagnosis-Related Group
AKI	Acute Kidney Injury
CAD	Coronary Artery Disease
CHF	Congestive Heart Failure
CKD	Chronic Kidney Disease
ICD-10	International Classification of Diseases, 10th Revision
LOS	Length of Stay
MATLAB	Matrix Laboratory
MI	Myocardial Infarction
NIS	National Inpatient Sample
OA	Osteoarthritis
OR	Odds Ratio
PE	Pulmonary Embolism
SPSS	Statistical Package for the Social Sciences
THA	Total Hip Arthroplasty
